# Supplementary material for: Human Belief State-Based Exploration and Exploitation in an Information-Selective Symmetric Reversal Bandit Task
Source: Comput Brain Behav. 2021 Aug 2;4(4):442–62. doi: 10.1007/s42113-021-00112-3 (PMC8327602; doi:10.1007/s42113-021-00112-3)
Supplement: Supplementary file 1 — (PDF 434 KB) [file 42113_2021_112_MOESM1_ESM.pdf]

Supplementary material

S.1. Sample characteristics

To characterize the group of participants, we measured symptoms of attention deficit hyperactivity disorder (ADHD), anxiety, depression and impulsivity. To this end, we used the questionnaires Conners Adult ADHD Rating Scale – Self Report, Short Version (CAARS-S:S; Conners et al. (1999)), State and Trait Anxiety Inventory (STAI; Spielberger et al. (1983)), Beck Depression Inventory II (BDI-II; Beck et al. (1996)) and UPPS-P Impulsive behavior Scale (Lynam et al., 2006), respectively. As shown in Table S.1, the sample varied only moderately with respect to these symptoms. For example, on CAARS-S:S, our main questionnaire of interest, participants scored within ±2 standard deviations of the mean of their age- and gender-matched norm groups of the general population. We therefore concluded that the sample represents the healthy population and did not relate individual variability in terms of ADHD or other clinical symptoms to behavioral strategies. We also report the IQ score, which was obtained by administering the Wechsler Abbreviated Scale of Intelligence (WASI-II; Wechsler (1999)) at the time of the Nathan Kline Institute Rockland Sample study (Nooner et al., 2012).

**Measurement**	**Range**	**Median**	**Mean ± SD**
Age (years)	18 - 35	23.5	24.5 ± 5.53
WASI-II (total score)	84 - 122	101.5	102.38 ± 9.14
CAARS-S:S (total T-score)	32 - 65	48	47.63 ± 9.16
BDI-II (total)	0 - 20	4	6.67 ± 6.3
STAI STATE (total T-score)	34 - 63	43	45.6 ± 8.52
STAI TRAIT (total T-score)	34 - 71	49.5	49.92 ± 10.29
UPPS-P (total)	73 - 190	118.5	124.04 ± 25.66

Table S.1. Sample characteristics.

S.2. Participant instructions

Participants were provided with the following task instructions:

Welcome to the main part of today's experiment! In the following we will introduce to you the decision making task that you will complete in the scanner. Please read the instructions carefully. If you have any questions, feel free to ask at any time. Once you read the instructions, you will complete a test run with the task to make sure you feel comfortable with it before going in the scanner. On every trial we will present to you two objects, an orange square and a blue triangle on either side of a black and grey screen and ask you to choose between them. One of these objects is profitable, meaning that it is going to give you a win most of the time, while the other object in not profitable meaning that it is going to give you a loss most of the time. Once you choose one of the objects, the outcome (win: +1 or loss: −1) will be registered to your account. You will have 2.5 seconds to indicate your choice. If you do not respond within this time window, the message 'Too slow' will appear on the screen and you automatically lose 1 point. Here you see an example for a trial (Supplementary Figure S.1).

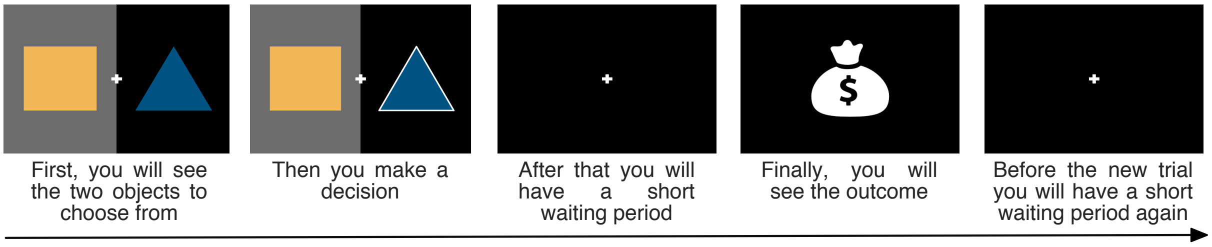

Figure S.1. Participant instructions 1. The figure shows the sequence of events within a trial as presented to the participants in the instructions.

You will start the experiment with a balance of 0 points and any wins or losses will be registered to your account. After the experiment, in addition to your standard payment for participation, you will receive up to $30 depending on your final account. Note that your balance cannot get below 0 and if you do not earn additional money on the task, you will not be penalized and you will still receive the standard payment for your participation. We would however encourage you to try to earn as much as possible on the task. After each run we will show you your balance. A run consists of 80 trials, which takes about 20 minutes to complete. You will have two runs in the scanner.

As mentioned above, one of the objects is profitable and it will bring you a win most of the times and every now and then it will bring you loss. At the same time, the other object is not profitable and it will bring you a loss most of the time and a win every now and then. You won't explicitly know which object is the profitable one and which is the non-profitable and you will need to conclude it from the outcomes. But be aware! These roles can switch, which means that the previously profitable object becomes non-profitable and the previously non-profitable object becomes profitable. Such a switch will happen only 1-4 times in the entire run and you will have enough trials without a switch to conclude which object is the profitable one.

Keep in mind that even the currently profitable object can from time to time deliver a loss and a couple of negative outcomes does not necessarily mean that a switch occurred. Similarly, even the non-profitable object can from time to time deliver a win and a couple of positive outcomes does not necessarily mean that a switch occurred. You can however assume that a switch has happened if you feel the previously rewarding object started to give you more losses than wins and the previously non-rewarding object started to bring you more wins than losses.

Before you do the test run, there is one more important aspect to the task: On each trial, one of the objects will be presented to you in front of a black background while the other object will be in front of a grey background. If you choose the object on the black-side, you will see the outcome of your choice. However, if you choose the grey-side object, the outcome will remain hidden from you but it will be registered to your account (Supplementary Figure S.2).

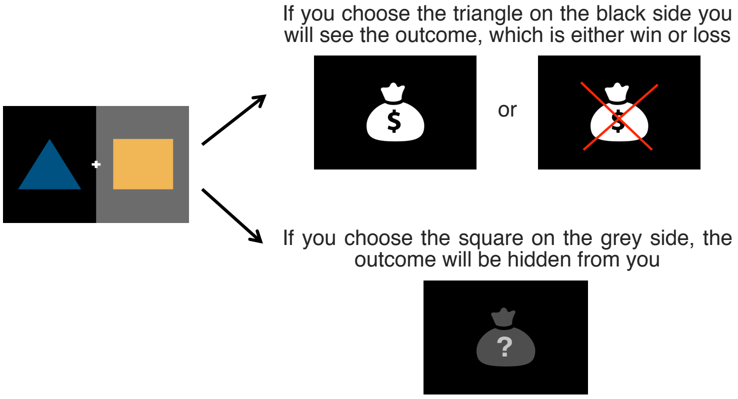

Figure S.2. Participant instructions 2. The figure depicts the lucrativeness and informativeness associated with the actions as presented to the participants in the instructions.

You will now complete a test run, which will be just like the ones you will complete in the scanner. We will discuss all your questions to make sure you feel comfortable with the task before going in the scanner.

Note that from the perspective of the participants the specification of the available actions was doubly over-specified: on the one hand, the square and the triangle were also colored orange and blue, and on the other hand, one side of the screen was also indicated by a black background, while the other side was indicated by a gray background. For efficiency, in the main text, we only retain the the notions of squares and triangles for available rewarded actions, black and grey backgrounds for informative and non-informative actions, and we use colors to indicate the currently lucrative (yellow) and detrimental (blue) actions.

S.3. Experimental state sequence

Table S.2 displays the definition of the state evolution function f. The upper table displays the state sequence of the first experimental run, the lower table displays the state sequence of the second run. t indexes the trial number and s_t is the trial state.

t	1	2	3	4	5	6	7	8	9	10
s_t	$(1,1)$	$(1,1)$	$(1,2)$	$(1,1)$	$(1,2)$	$(1,1)$	$(1,1)$	$(1,2)$	$(1,1)$	$(1,1)$

t	11	12	13	14	15	16	17	18	19	20
s_t	$(1,1)$	$(1,1)$	$(1,2)$	$(1,2)$	$(1,2)$	$(1,1)$	$(1,2)$	$(1,2)$	$(1,2)$	$(2,1)$

t	21	22	23	24	25	26	27	28	29	30
s_t	$(2,1)$	$(2,2)$	$(2,1)$	$(2,2)$	$(2,1)$	$(2,2)$	$(2,2)$	$(2,1)$	$(2,2)$	$(2,2)$

t	31	32	33	34	35	36	37	38	39	40
s_t	$(2,1)$	$(2,1)$	$(2,2)$	$(2,1)$	$(2,2)$	$(2,1)$	$(2,2)$	$(2,1)$	$(2,2)$	$(2,1)$

t	41	42	43	44	45	46	47	48	49	50
s_t	$(2,2)$	$(2,2)$	$(1,2)$	$(1,2)$	$(1,1)$	$(1,1)$	$(1,2)$	$(1,2)$	$(1,2)$	$(1,2)$

t	51	52	53	54	55	56	57	58	59	60
s_t	$(1,1)$	$(1,2)$	$(1,1)$	$(1,2)$	$(1,2)$	$(1,1)$	$(1,1)$	$(1,2)$	$(1,1)$	$(1,1)$

t	61	62	63	64	65	66	67	68	69	70
s_t	$(1,1)$	$(1,1)$	$(1,2)$	$(2,1)$	$(2,1)$	$(2,2)$	$(2,1)$	$(2,2)$	$(2,1)$	$(2,1)$

t	71	72	73	74	75	76	77	78	79	80
s_t	$(2,2)$	$(2,1)$	$(2,1)$	$(2,1)$	$(2,2)$	$(2,2)$	$(2,2)$	$(2,2)$	$(2,2)$	$(2,1)$

t	1	2	3	4	5	6	7	8	9	10
s_t	$(2,1)$	$(2,1)$	$(2,1)$	$(2,2)$	$(2,1)$	$(2,1)$	$(2,1)$	$(2,1)$	$(2,2)$	$(2,2)$

t	11	12	13	14	15	16	17	18	19	20
s_t	$(2,1)$	$(2,1)$	$(2,2)$	$(2,2)$	$(2,1)$	$(2,2)$	$(2,2)$	$(2,2)$	$(2,2)$	$(2,1)$

t	21	22	23	24	25	26	27	28	29	30
s_t	$(2,1)$	$(2,1)$	$(2,2)$	$(1,1)$	$(1,2)$	$(1,2)$	$(1,2)$	$(1,1)$	$(1,2)$	$(1,1)$

t	31	32	33	34	35	36	37	38	39	40
s_t	$(1,2)$	$(1,2)$	$(1,2)$	$(1,2)$	$(1,2)$	$(1,1)$	$(1,1)$	$(1,2)$	$(1,2)$	$(1,2)$

t	41	42	43	44	45	46	47	48	49	50
s_t	$(1,2)$	$(1,1)$	$(2,2)$	$(2,2)$	$(2,2)$	$(2,1)$	$(2,2)$	$(2,2)$	$(2,2)$	$(2,1)$

t	51	52	53	54	55	56	57	58	59	60
s_t	$(2,2)$	$(2,1)$	$(2,2)$	$(2,1)$	$(2,1)$	$(2,2)$	$(2,1)$	$(2,2)$	$(2,1)$	$(1,2)$

t	61	62	63	64	65	66	67	68	69	70
s_t	$(1,1)$	$(1,1)$	$(1,2)$	$(1,1)$	$(1,1)$	$(1,2)$	$(1,1)$	$(1,1)$	$(1,1)$	$(1,1)$

t	71	72	73	74	75	76	77	78	79	80
s_t	$(1,2)$	$(1,1)$	$(1,1)$	$(1,2)$	$(1,1)$	$(1,2)$	$(1,1)$	$(1,1)$	$(1,1)$	$(1,2)$

Table S.2. Experimental state sequence.

S.4. Belief state, posterior predictive distribution, and KL-divergence

Belief state

Based on the probability distributions $p\left(s_1^1\right)$, $p\left(s_{t+1}^1|s_t^1\right)$ and $p^{a_t}\left(o_t|s_t^1\right)$, the agent's belief state on trials $t = 2, ..., T$ can be recursively evaluated according to eq. (15) of the main text. To show the validity of this equation, we first express the belief state as

$$p^{a_{1:t-1}}\left(s_t^1|o_{1:t-1}\right) = \frac{p^{a_{1:t-1}}\left(s_t^1, o_{1:t-1}\right)}{p^{a_{1:t-1}}\left(o_{1:t-1}\right)}. \tag{S.1}$$

The numerator of eq. (S.1) can then be rewritten as

$$\begin{aligned}
p^{a_{1:t-1}}\left(s_t^1, o_{1:t-1}\right) &= \sum_{s_{t-1}^1} p^{a_{1:t-1}}\left(s_t^1, s_{t-1}^1, o_{1:t-1}\right) \\
&= \sum_{s_{t-1}^1} p^{a_{1:t-1}}\left(s_t^1|s_{t-1}^1, o_{1:t-1}\right) p^{a_{1:t-1}}\left(s_{t-1}^1, o_{1:t-2}, o_{t-1}\right) \\
&= \sum_{s_{t-1}^1} p\left(s_t^1|s_{t-1}^1\right) p^{a_{t-2:t-1}}\left(o_{t-1}|s_{t-1}^1, o_{1:t-2}\right) p^{a_{1:t-2}}\left(s_{t-1}^1, o_{1:t-2}\right) \\
&= \sum_{s_{t-1}^1} p\left(s_t^1|s_{t-1}^1\right) p^{a_{t-1}}\left(o_{t-1}|s_{t-1}^1\right) p^{a_{1:t-2}}\left(s_{t-1}^1|o_{1:t-2}\right) p^{a_{1:t-2}}\left(o_{1:t-2}\right) \\
&= p^{a_{1:t-2}}\left(o_{1:t-2}\right) \sum_{s_{t-1}^1} p\left(s_t^1|s_{t-1}^1\right) p^{a_{t-1}}\left(o_{t-1}|s_{t-1}^1\right) p^{a_{1:t-2}}\left(s_{t-1}^1|o_{1:t-2}\right),
\end{aligned} \tag{S.2}$$

where we used the conditional independence of s_t^1 of $o_{1:t-1}$ given s_{t-1}^1 in the third equality and the conditional independence of o_{t-1} of all other random variables given s_{t-1}^1 in the fourth equality. Similarly, we can rewrite the denominator of eq. (S.1) as

$$\begin{aligned}
p^{a_{1:t-1}}\left(o_{1:t-1}\right) &= \sum_{s_t^1} \sum_{s_{t-1}^1} p^{a_{1:t-1}}\left(s_t^1, s_{t-1}^1, o_{1:t-1}\right) \\
&= p^{a_{1:t-2}}\left(o_{1:t-2}\right) \sum_{s_t^1} \sum_{s_{t-1}^1} p\left(s_t^1|s_{t-1}^1\right) p^{a_{t-1}}\left(o_{t-1}|s_{t-1}^1\right) p^{a_{1:t-2}}\left(s_{t-1}^1|o_{1:t-2}\right),
\end{aligned} \tag{S.3}$$

where in the last equality we used the numerator's form derived in eq. (S.2). Finally, by substitution of (S.2) and (S.3) in (S.1), we obtain the belief state update formula of eq. (15) in the main text as follows:

$$\begin{aligned}
p^{a_{1:t-1}}\left(s_t^1|o_{1:t-1}\right) &= \frac{p^{a_{1:t-2}}\left(o_{1:t-2}\right) \sum_{s_{t-1}^1} p\left(s_t^1|s_{t-1}^1\right) p^{a_{t-1}}\left(o_{t-1}|s_{t-1}^1\right) p^{a_{1:t-2}}\left(s_{t-1}^1|o_{1:t-2}\right)}{p^{a_{1:t-2}}\left(o_{1:t-2}\right) \sum_{s_t^1} \sum_{s_{t-1}^1} p\left(s_t^1|s_{t-1}^1\right) p^{a_{t-1}}\left(o_{t-1}|s_{t-1}^1\right) p^{a_{1:t-2}}\left(s_{t-1}^1|o_{1:t-2}\right)} \\
&= \frac{\sum_{s_{t-1}^1} p\left(s_t^1|s_{t-1}^1\right) p^{a_{t-1}}\left(o_{t-1}|s_{t-1}^1\right) p^{a_{1:t-2}}\left(s_{t-1}^1|o_{1:t-2}\right)}{\sum_{s_t^1} \sum_{s_{t-1}^1} p\left(s_t^1|s_{t-1}^1\right) p^{a_{t-1}}\left(o_{t-1}|s_{t-1}^1\right) p^{a_{1:t-2}}\left(s_{t-1}^1|o_{1:t-2}\right)}.
\end{aligned} \tag{S.4}$$

Posterior predictive distribution

Given the agent's belief state $p^{a_{1:t-1}}\left(s_t^1|o_{1:t-1}\right)$ and the action-dependent state-conditional observation distribution $p^{a_t}\left(o_t|s_t^1\right)$, the posterior predictive distribution can be evaluated according to eq. (23) of the main text. A proof of this equation is as follows:

$$
\begin{aligned}
p^{a_{1:t}}\left(o_t|o_{1:t-1}\right) &= \frac{p^{a_{1:t}}\left(o_t, o_{1:t-1}\right)}{p^{a_{1:t}}\left(o_{1:t-1}\right)} \\
&= \frac{\sum_{s_t^1} p^{a_{1:t}}\left(o_t, o_{1:t-1}, s_t^1\right)}{p^{a_{1:t}}\left(o_{1:t-1}\right)} \\
&= \frac{\sum_{s_t^1} p^{a_{1:t}}\left(s_t^1|o_t, o_{1:t-1}\right) p^{a_{1:t}}\left(o_t, o_{1:t-1}\right)}{p^{a_{1:t}}\left(o_{1:t-1}\right)} \\
&= \frac{\sum_{s_t^1} p^{a_{1:t-1}}\left(s_t^1|o_{1:t-1}\right) \sum_{s_t^1} p^{a_{1:t}}\left(o_t, o_{1:t-1}, s_t^1\right)}{p^{a_{1:t}}\left(o_{1:t-1}\right)} \\
&= \frac{\sum_{s_t^1} p^{a_{1:t-1}}\left(s_t^1|o_{1:t-1}\right) p^{a_{1:t}}\left(o_t|o_{1:t-1}, s_t^1\right) p^{a_{1:t}}\left(o_{1:t-1}, s_t^1\right)}{\sum_{s_t^1} p^{a_{1:t}}\left(o_{1:t-1}, s_t^1\right)} \\
&= \sum_{s_t^1} p^{a_{1:t-1}}\left(s_t^1|o_{1:t-1}\right) p^{a_t}\left(o_t|s_t^1\right) \\
&= b_t p^{a_t}\left(o_t|s_t^1 = 1\right) + (1 - b_t)\, p^{a_t}\left(o_t|s_t^1 = 2\right), \qquad \text{(S.5)}
\end{aligned}
$$

where in the last equality we substituted the belief state with its scalar representation.

KL-divergence

Recall that the KL-divergence for two distributions p and q of a discrete random variable x is defined as (Kullback & Leibler, 1951)

$$\mathrm{KL}\left(p(x)||q(x)\right) = \sum_{x \in \mathcal{X}} p(x) \ln\left(\frac{p(x)}{q(x)}\right). \qquad \text{(S.6)}$$

With

$$b := p^{a_{1:t-1}}(s_t^1 = 1|o_{1:t-1}) \qquad \text{(S.7)}$$

and thus

$$p^{a_{1:t-1}}(s_t^1 = 2|o_{1:t-1}) = 1 - b, \qquad \text{(S.8)}$$

as well as

$$b^{a,o} := p^{a_{1:t-1}, a_t = a}(s_{t+1}^1 = 1|o_{1:t-1}, o_t = o) \qquad \text{(S.9)}$$

and thus

$$p^{a_{1:t-1}, a_t = a}(s_{t+1}^1 = 2|o_{1:t-1}, o_t = o) = 1 - b^{a,o} \qquad \text{(S.10)}$$

we have

$$
\begin{aligned}
&\mathrm{KL}\left(p^{a_{1:t-1},a_t=a}(s^1_{t+1}|o_{1:t-1},o_t=o)||p^{a_{1:t-1}}(s^1_t|o_{1:t-1})\right) \\
&= \sum_{s^1\in S^1} p^{a_{1:t-1},a_t=a}(s^1_{t+1}|o_{1:t-1},o_t=o)\ln\left(\frac{p^{a_{1:t-1},a_t=a}(s^1_{t+1}|o_{1:t-1},o_t=o)}{p^{a_{1:t-1}}(s^1_t|o_{1:t-1})}\right) \\
&= \quad p^{a_{1:t-1},a_t=a}(s^1_{t+1}=1|o_{1:t-1},o_t=o)\ln\left(\frac{p^{a_{1:t-1},a_t=a}(s^1_{t+1}=1|o_{1:t-1},o_t=o)}{p^{a_{1:t-1}}(s^1_t=1|o_{1:t-1})}\right) \\
&\quad + p^{a_{1:t-1},a_t=a}(s^1_{t+1}=2|o_{1:t-1},o_t=o)\ln\left(\frac{p^{a_{1:t-1},a_t=a}(s^1_{t+1}=2|o_{1:t-1},o_t=o)}{p^{a_{1:t-1}}(s^1_t=2|o_{1:t-1})}\right) \\
&= (1-b^{a,o})\ln\left(\frac{1-b^{a,o}}{1-b}\right)+b^{a,o}\ln\left(\frac{b^{a,o}}{b}\right).
\end{aligned}
\tag{S.11}
$$

S.5. Belief state and posterior predictive distribution implementation

For a concise implementation of the belief state update and the posterior predictive distribution formulas, we represent the agent's probability distributions by stochastic vectors and stochastic matrices. Specifically, in the implementation of the agent model components as defined in *irb_modcomp.m*,

- $\mu_1 \in \mathbb{R}_{\geq 0}^{|S^1|}$ represents the initial belief state $p\left(s_1^1\right)$. The ith entry of μ_1 corresponds to the agent's subjective uncertainty that the non-observable state component takes on value $s_1^1 = i$ at trial $t = 1$. Formally,

$$\mu_1 := \begin{pmatrix} p\left(s_1^1 = 1\right) \\ p\left(s_1^1 = 2\right) \end{pmatrix} = \begin{pmatrix} 0.5 \\ 0.5 \end{pmatrix}. \tag{S.12}$$

- $\mu_t \in \mathbb{R}_{\geq 0}^{|S^1|}$ represents the belief state $p^{a_{1:t-1}}\left(s_t^1|o_{1:t-1}\right)$ at trial t. The ith entry of μ_t corresponds to the agent's subjective uncertainty that the non-observable state component takes on value $s_t^1 = i$ at trial t given the history of observations $o_{1:t-1}$ and actions $a_{1:t-1}$. Formally,

$$\mu_t := \begin{pmatrix} p^{a_{1:t-1}}\left(s_t^1 = 1|o_{1:t-1}\right) \\ p^{a_{1:t-1}}\left(s_t^1 = 2|o_{1:t-1}\right) \end{pmatrix}. \tag{S.13}$$

- $\Phi \in \mathbb{R}_{\geq 0}^{|S^1|\times|S^1|}$ represents the state-state transition distribution $p\left(s_{t+1}^1|s_t^1\right)$. The jth entry of the ith row of Φ corresponds to the agent's subjective uncertainty that the non-observable state component takes on the value $s_{t+1}^1 = j$ in trial $t+1$ given that $s_t^1 = i$ in trial t. Formally,

$$\Phi := \begin{pmatrix} p\left(s_{t+1}^1 = 1|s_t^1 = 1\right) & p\left(s_{t+1}^1 = 2|s_t^1 = 1\right) \\ p\left(s_{t+1}^1 = 1|s_t^1 = 2\right) & p\left(s_{t+1}^1 = 2|s_t^1 = 2\right) \end{pmatrix} = \begin{pmatrix} 0.9625 & 0.0375 \\ 0.0375 & 0.9625 \end{pmatrix}. \tag{S.14}$$

- $\Omega^{a_t} \in \mathbb{R}_{\geq 0}^{|S^1|\times|O|}$ represents the action-dependent state-conditional observation distribution $p^{a_t}\left(o_t|s_t^1\right)$ for action $a \in A$. The kth entry of the ith row of $\Omega^{a_t=a}$ corresponds to the agent's subjective uncertainty that the observation takes on the value $o_t = k$ given that the non-observable state component takes on the value $s_t^1 = i$ and the action value is $a_t = a$. Formally, for the informative actions

$$\Omega^{a_t=1} := \begin{pmatrix} p^1\left(o_t = 1|s_t^1 = 1\right) & p^1\left(o_t = 2|s_t^1 = 1\right) & p^1\left(o_t = 3|s_t^1 = 1\right) \\ p^1\left(o_t = 1|s_t^1 = 2\right) & p^1\left(o_t = 2|s_t^1 = 2\right) & p^1\left(o_t = 3|s_t^1 = 2\right) \end{pmatrix} = \begin{pmatrix} 0.15 & 0.85 & 0 \\ 0.85 & 0.15 & 0 \end{pmatrix}, \tag{S.15}$$

and

$$\Omega^{a_t=3} := \begin{pmatrix} p^3\left(o_t = 1|s_t^1 = 1\right) & p^3\left(o_t = 2|s_t^1 = 1\right) & p^3\left(o_t = 3|s_t^1 = 1\right) \\ p^3\left(o_t = 1|s_t^1 = 2\right) & p^3\left(o_t = 2|s_t^1 = 2\right) & p^3\left(o_t = 3|s_t^1 = 2\right) \end{pmatrix} = \begin{pmatrix} 0.85 & 0.15 & 0 \\ 0.15 & 0.85 & 0 \end{pmatrix}, \tag{S.16}$$

and for the non-informative actions

$$\Omega^{a_t\in\{2,4\}} := \begin{pmatrix} p^{a_t}\left(o_t = 1|s_t^1 = 1\right) & p^{a_t}\left(o_t = 2|s_t^1 = 1\right) & p^{a_t}\left(o_t = 3|s_t^1 = 1\right) \\ p^{a_t}\left(o_t = 1|s_t^1 = 2\right) & p^{a_t}\left(o_t = 2|s_t^1 = 2\right) & p^{a_t}\left(o_t = 3|s_t^1 = 2\right) \end{pmatrix} = \begin{pmatrix} 0 & 0 & 1 \\ 0 & 0 & 1 \end{pmatrix}. \tag{S.17}$$

- $\Psi^{a_t} \in \mathbb{R}_{\geq 0}^{|S^1|\times|R|}$ represents the action-dependent state-conditional reward distribution $p^{a_t}\left(r_t|s_t^1\right)$ for action $a \in A$. The lth entry of the ith row of $\Psi^{a_t=a}$ corresponds to the agent's subjective uncertainty that the reward takes on the value $r_t = l - m$ given that the non-observable state component takes on the value $s_t^1 = i$ and action the value $a_t = a$. Note that m is introduced

to convert the linear indices to reward values and takes on the value 2 if $l = 1$ and the value 1 if $l = 2$. Formally,

$$\Psi^{a_t \in \{1,2\}} := \begin{pmatrix} p^{a_t}\left(r_t = -1 | s_t^1 = 1\right) & p^{a_t}\left(r_t = +1 | s_t^1 = 1\right) \\ p^{a_t}\left(r_t = -1 | s_t^1 = 2\right) & p^{a_t}\left(r_t = +1 | s_t^1 = 2\right) \end{pmatrix} = \begin{pmatrix} 0.15 & 0.85 \\ 0.85 & 0.15 \end{pmatrix} \tag{S.18}$$

represents the action-dependent state-conditional reward distribution for the actions of choosing the square, and

$$\Psi^{a_t \in \{3,4\}} := \begin{pmatrix} p^{a_t}\left(r_t = -1 | s_t^1 = 1\right) & p^{a_t}\left(r_t = +1 | s_t^1 = 1\right) \\ p^{a_t}\left(r_t = -1 | s_t^1 = 2\right) & p^{a_t}\left(r_t = +1 | s_t^1 = 2\right) \end{pmatrix} := \begin{pmatrix} 0.85 & 0.15 \\ 0.15 & 0.85 \end{pmatrix} \tag{S.19}$$

represents the action-dependent state-conditional reward distribution for the actions of choosing the triangle. Accordingly, $\sum_{l=1}^{|R|} \Psi^{a_t}_{i(l-m)} = 1$.

Based on the definitions above and using the standard matrix product $\cdot$ as well as the element-wise (Hadamard) matrix product $\circ$, the agent's belief state at trial t (eq. (15) of the main text) can be written as

$$\mu_t := \tilde{\mu}_t \cdot \left(\sum_{i=1}^{|S^1|} \tilde{\mu}_{t_i} \right)^{-1}, \tag{S.20}$$

where

$$\tilde{\mu}_t := \Phi \cdot \left(\Omega_k^a \circ \mu_{t-1}\right) \tag{S.21}$$

is the unnormalized belief state following action $a_{t-1} = a$ and observation $o_{t-1} = k$ and $\left(\sum_{i=1}^{|S^1|} \tilde{\mu}_{t_i}\right)^{-1}$ is the normalization constant. Here, Ω_k^a denotes the kth column of Ω^a and μ_{t-1} denotes the prior belief state on trial $t-1$, which corresponds to eq. (S.13), if $t-1 > 1$, and to eq. (S.12), if $t-1 = 1$. Similarly, the posterior predictive distribution (eq. (23) of the main text) for action $a_t = a$ can be written as

$$\omega_t := \left(\Omega^a\right)^T \cdot \mu_t. \tag{S.22}$$

References

Beck, A. T., Steer, R. A., & Brown, G. K. (1996). Beck Depression Inventory-II. *San Antonio University Press.*

Conners, C. K., Erhardt, D., & Sparrow, E. (1999). CAARS. Adult ADHD rating scales. *Technical Manual.*

Lynam, D. R., Smith, G. T., Whiteside, S. P., & Cyders, M. A. (2006). The UPPS-P: Assessing five personality pathways to impulsive behavior. *Purdue University Press.*

Nooner, K. B., Colcombe, S., Tobe, R., Mennes, M., Benedict, M., Moreno, A., Panek, L., Brown, S., Zavitz, S., Li, Q., et al. (2012). The NKI-Rockland sample: A model for accelerating the pace of discovery science in psychiatry. *Frontiers in Neuroscience*, 6, 152.

Spielberger, C., Gorsuch, R., Lushene, R., Vagg, P., & Jacobs, G. (1983). Manual for the state-trait anxiety inventory. *Consulting Psychologists Press.*

Wechsler, D. (1999). Wechsler Abbreviated Scale of Intelligence. *The Psychological Corporation: Harcourt Brace & Company.*

Kullback, S. & Leibler, R. A. (1951-03, 1951). On information and sufficiency. *The Annals of Mathematical Statistics*, 22(1), 79–86.
